# Amber (Succinite) Extract Enhances Glucose Uptake through the Up-Regulation of ATP and Down-Regulation of ROS in Mouse C2C12 Cells

**DOI:** 10.3390/ph17050586

**Published:** 2024-05-03

**Authors:** Mahmoud Ben Othman, Reiko Takeda, Marie Sekita, Kazuma Okazaki, Kazuichi Sakamoto

**Affiliations:** 1 Graduate School of Life and Environmental Sciences, University of Tsukuba, Tsukuba 305-8572, Japan; sakazbo@gmail.com; 2 Kohaku Bio Technology Co., Ltd., Morioka 020-8551, Japan; r-takeda@yamanobeautychemical.com (R.T.); m-sekita@yamanobeautychemical.com (M.S.); k-okazaki@yamanobeautychemical.com (K.O.)

**Keywords:** amber, C2C12 cells, glucose uptake, ROS, ATP, *GLUT1*, *GLUT4*

## Abstract

Traditionally, amber (Succinite) has been used to alleviate all types of pain, skin allergies, and headaches. However, no studies have been conducted on its antidiabetic and antioxidant effects. In this study, differentiated skeletal muscle C2C12 cells were used to demonstrate the protective effects of amber (AMB) against H_2_O_2_-induced cell death. In addition, the effects of AMB on glucose uptake and ATP production were investigated. Our results showed that AMB at 10, 25, and 50 μg/mL suppressed the elevation of ROS production induced by H_2_O_2_ in a dose-dependent manner. Moreover, AMB enhanced glucose utilization in C2C12 cells through the improvement of ATP production and an increase in PGC-1α gene expression resulting in an amelioration of mitochondrial activity. On the other hand, AMB significantly increased the gene expression of glucose transporters *GLUT4* and *GLUT1*. Our finding suggests that AMB can be used as a natural supplement for diabetes treatment and for the promotion of skeletal muscle function.

## 1. Introduction

Skeletal muscle is an elastic and amenable tissue that represents 40% of total body mass and it is considered the major site for human energy production, which is responsible for approximately 70–80% of the glucose uptake and oxidation in the body [1,2]. In addition, skeletal muscle is known to play diverse crucial physical and metabolic roles in humans, including maintaining body homeostasis, preventing excess movement, and generating movement [3].

Myogenesis is the process of generating skeletal muscle, which is characterized by the proliferation and differentiation of myoblasts into muscle cells. This process results in the fusion of muscle cells into mature myotubes, which is guaranteed by the expression of muscle-specific genes, cytokines, and various growth factors [4]. In addition, myogenesis is essential for skeletal muscle regeneration, development, and maintenance.

Hyperglycemia is a condition in which an excessive amount of glucose circulates in the blood plasma. Chronic hyperglycemia can result in dysfunction and damage, such as heart and blood vessel disease and weakness of the tissue of the pancreas, kidney, and liver, leading to the development of Diabetes Mellitus (DM), which is the seventh leading cause of death, and it is projected to affect 591.9 million people by 2035 [5]. To prevent or diminish hyperglycemia, the human body stimulates glucose uptake. In skeletal muscles, glucose uptake is mediated via two main pathways: an insulin-dependent pathway, in which insulin stimulates some signaling pathways including protein kinase B (AKT), phosphoinositide 3-kinase (PI3K), and *GLUT4* translocation; and an insulin-independent pathway represented by 5′-adenosine monophosphate-activated protein kinase (AMPK), which is an enzyme that plays a role in cellular energy homeostasis, mainly in activating glucose and fatty acid uptake [6]. One of the main factors reducing glucose uptake in skeletal muscles is chronic oxidative stress. Reactive oxygen species (ROS) have been demonstrated as an important regulator of signal transduction and to increase glucose uptake and insulin sensitivity in skeletal muscle [7]. On the other hand, Lipko and Debski [8] reported that a low concentration of H_2_O_2_ is necessary for glucose transport stimulation and *GLUT4* translocation in skeletal muscle cells. However, it was demonstrated that chronic treatment with H_2_O_2_ reduces insulin-dependent Akt2 phosphorylation and *GLUT4* translocation [9].

Nowadays, the use of natural products from plants for the treatment of several disorders is becoming an alternative due to their efficacy and minimal side effects [10]. Amber (AMB) is a fossilized tree resin derived from different types of conifers and consists of organic polymers formed through complex maturation processes of the original plant resin over millions of years. It has been used as medicine for muscle pain, skin allergies, and headaches [11]. Our laboratory group has demonstrated that AMB could decrease the triacylglycerol, glucose uptake, and leptin in 3T3-L1 cells [12].

Differentiated C2C12 muscle cells were used in this study as a model to evaluate the protective effects of an ethanol extract of amber against H_2_O_2_-induced ATP depletion, oxidative stress, and decreases in glucose uptake and mitochondrial biogenesis-related genes such as *GLUT4*, *GLUT1*, and *PGC-1α.*

## 2. Results

### 2.1. Protective Effect of AMB against H_2_O_2_

**Figure 1:**
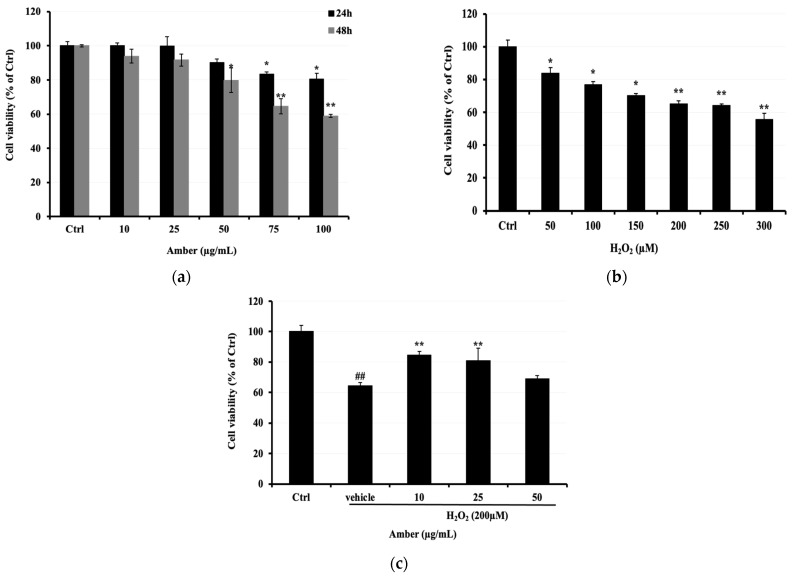
Protective properties of AMB against H_2_O_2_-induced C2C12 cell cytotoxicity. C2C12 cells were seeded at 0.5 × 10^4^ cells/well in a 96-well microplate and treated with (**a**) AMB (10, 25, 50, 75, and 100 µg/mL) for 24 and 48 h; (**b**) C2C12 cells were treated with H_2_O_2_ (50, 100, 150, 200, 250, and 300 µM); (**c**) C2C12 cells were pretreated with AMB for 24 h and then treated with 200 µM H_2_O_2_ for another 24 h. Cell viability was determined using an MTT assay as explained in the Materials and Methods. Each bar represents the mean of 3 independent trials ± SD. ## indicate a significant difference (*p* < 0.01) versus untreated control group; * *p* < 0.05, ** indicate a significant difference (*p* < 0.01) versus vehicle group (Student’s *t*-test).

First, we assessed the cytotoxicity of different concentrations of AMB (0, 10, 25, 50, 75, and 100 μg/mL) on C2C12 cells. It was observed that AMB displayed a significant cytotoxic effect at the highest doses of 75 and 100 μg/mL, reaching 83% and 80%, respectively (Figure 1a). Thus, we determined the cell viability of C2C12 cells exposed to H_2_O_2_ (0–300 μM). Based on the results shown in Figure 1b, the concentration of 200 μM was selected to investigate the protective effect of AMB on H_2_O_2_-treated cells. The cell viability was observed to be 84.42, 80.78, and 69.10% in C2C12 cells pretreated with AMB at 10, 25, and 50 μg/mL, respectively, before H_2_O_2_ exposure. Cells treated with H_2_O_2_ only at 200 μM showed a viability of 64.27% (Figure 1c).

### 2.2. ROS Production in AMB-Treated C2C12 Cells

**Figure 2:**
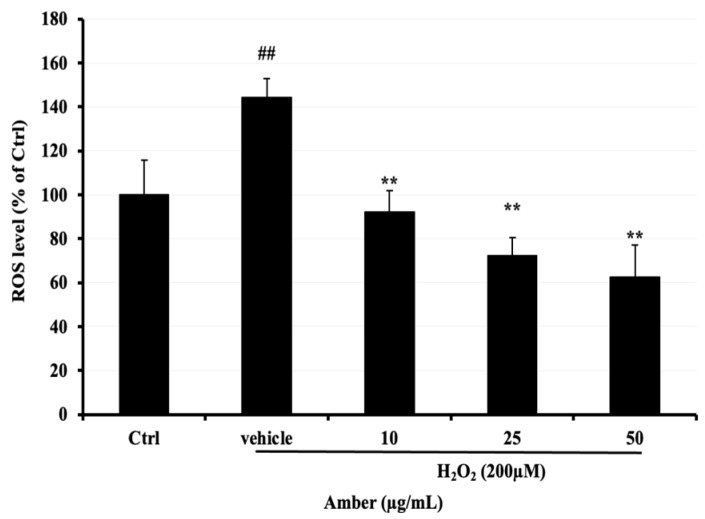
Effect of AMB on H_2_O_2_-induced ROS elevation in C2C12 cells. After 5 days of differentiation, C2C12 cells were treated with amber (10, 25, and 50 µg/mL) for 24 h and then exposed to 200 µM of H_2_O_2_ for 3 h. Asterisks indicate a significant difference (*p* < 0.01) compared with the vehicle-only control group (cells treated with H_2_O_2_ only). The hash indicates a significant difference (*p* < 0.01) compared with the control (Ctrl) group. The results represent three independent experiments; each experiment contained triplicate samples.

Next, 2, 7-dichlorodihydrofluorescein (DCFH-DA), a fluorescent dye, was used to compare ROS levels in C2C12 cells after treatment with AMB at 10, 25, and 50 μg/mL compared with the H_2_O_2_-treated group, ROS levels were significantly increased in the H_2_O_2_-treated group compared to the control group, reaching approximately 140%. However, the ROS levels in AMB-treated groups were significantly decreased, reaching 29% and 39% at 25 and 50 μg/mL, respectively (Figure 2).

### 2.3. Effect of AMB on Glucose Uptake in C2C12 Cells

**Figure 3:**
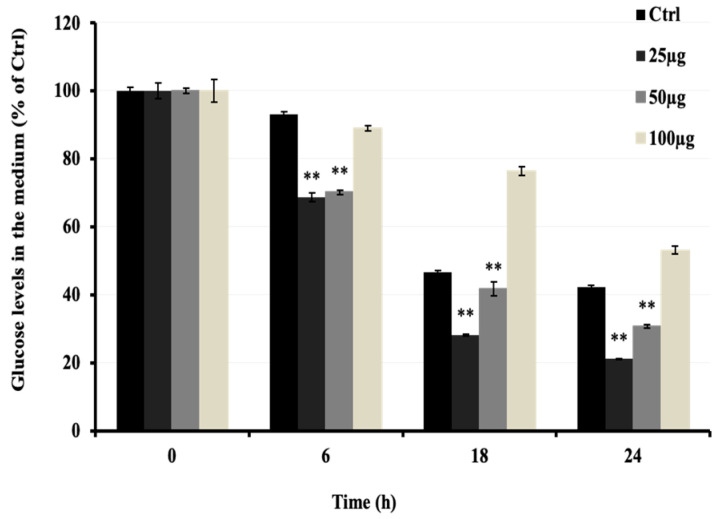
Effects of AMB on glucose uptake in C2C12 cells. Cells were seeded at 3 × 10^4^ cells/well in a 24-well microplate. After 48 h, the DMEM medium was changed to 2% HS medium to start differentiation for 5 days. Then, a fresh medium containing AMB at 10, 25, and 50 µg/mL was added to cells for 24 h. Asterisks indicate a significant difference (*p* < 0.01) compared with the control (Ctrl) group at each time of sampling. The results represent three independent experiments; each experiment contained triplicate samples.

To investigate the anti-hyperglycemic potential of AMB, the increasing glucose uptake effect was investigated in C2C12 cells in vitro. As shown in Figure 3, AMB at 25 and 50 μg/mL significantly decreased the glucose levels in the medium in a time- but not a dose-dependent manner. Compared to the control group, the low dose of AMB showed a stronger effect on glucose uptake (50%) than the high dose (30%).

### 2.4. Effect of AMB on ATP Levels in C2C12 Cells

**Figure 4:**
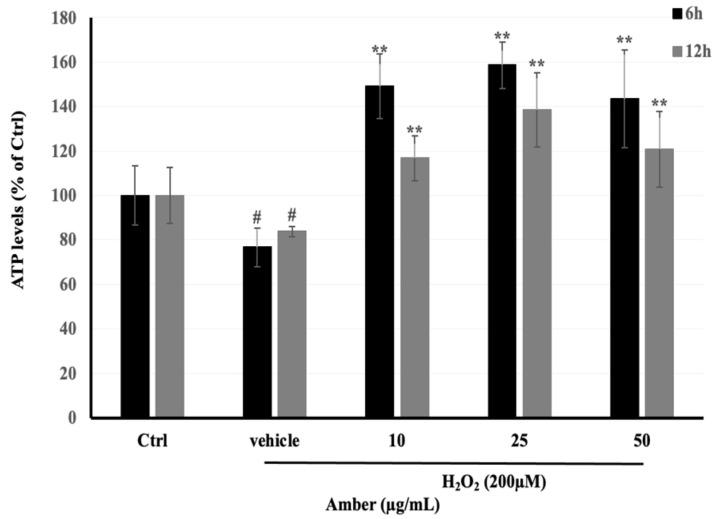
Effects of H_2_O_2_-induced ATP depletion in C2C12 cells incubated with AMB extract (10, 25, and 50 µg/mL) for 24 h and then exposed to 200 µM of H_2_O_2_ for 3 h. Asterisks indicate a significant difference (*p* < 0.01) compared with the vehicle-only control group (cells treated with H_2_O_2_ only). Hash indicates a significant difference (*p* < 0.05) compared with the control (Ctrl) group. The results represent three independent experiments; each experiment contained triplicate samples.

ATP levels in C2C12 cells treated with AMB were measured with an ATP Colorimetric/Fluorometric Assay Kit (TOYO INK GROUP, Tokyo, Japan) according to the manufacturer’s instructions. From the results, we found that ATP levels increased in AMB groups in both times of treatment, 6 and 12 h (Figure 4). In addition, the low dose of AMB (10 μg/mL) showed the highest effect on ATP production, which reached 206% and 142% after 6 and 12 h of treatment, respectively. In addition, the ATP levels were decreased in the H_2_O_2_-treated group, reaching 80% compared to the control group.

### 2.5. Effect of AMB on the Copy Number of Mitochondria in C2C12 Cells

**Figure 5:**
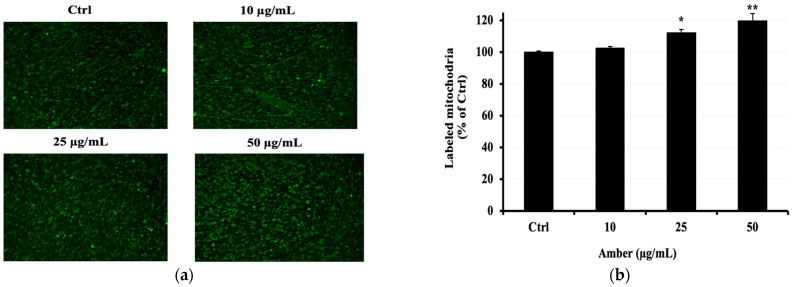
Effects of AMB on the mitochondrial activity in C2C12 cells. Differentiated C2C12 cells were cultured with AMB (10, 25, and 50 µg/mL) for 48 h. The mitochondrial number was measured by fluorescent staining. (**a**) The mitochondria are indicated in green; images were captured at 10 magnifications using a fluorescence microscope. (**b**) Fluorescence intensity. Asterisks indicate a significant difference (* *p* < 0.05, ** *p* < 0.01) compared with the control group.

To confirm whether the AMB extract increases mitochondrial activity and mass, we performed mitochondria staining. As shown in Figure 5a, AMB enhanced mitochondrial fluorescence intensity in C2C12 myotubes at 25 and 50 μg/mL doses compared to the control group.

### 2.6. Effect of AMB on GLUT4, GLUT1, and PGC-1α Gene Expression in C2C12 Cells

**Figure 6:**
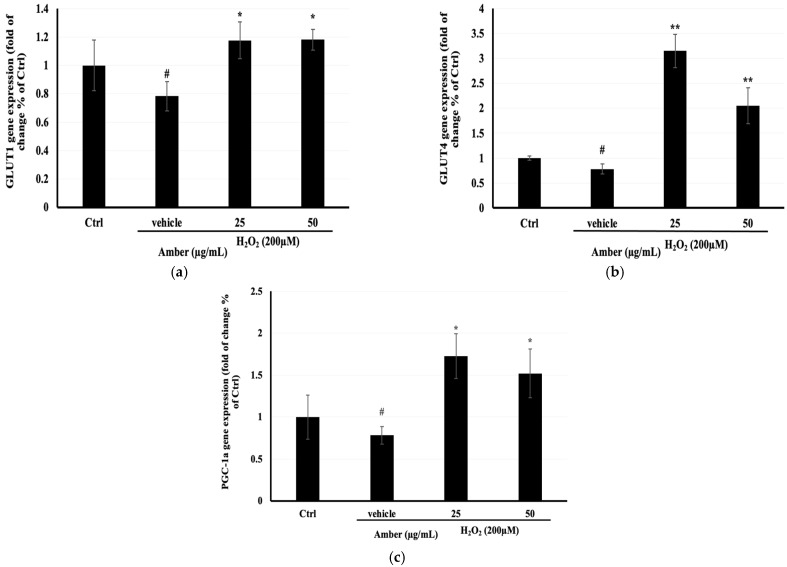
Effects of AMB on mRNA expression of (**a**) *Glut1*, (**b**) *Glut4,* and (**c**) *PGC-1α* in C2C12 cells. C2C12 cells were seeded at 20 × 10^4^ cells/well in a 6 cm dish and were treated with amber (25 and 50 µg/mL) after 7 days of differentiation, for 24 h, and then they were treated with 200 µM H_2_O_2_ for 6 h. The mRNA expression of genes was normalized to *GAPDH* mRNA expression and was expressed as a ratio compared to the control. Each bar represents the mean of duplicate ± SD. * *p* < 0.05, ** *p* < 0.01 versus the vehicle group, and # *p* < 0.05 versus the control group (Student’s *t*-test).

To better understand the mechanism behind the increase in glucose uptake and mitochondrial mass in C2C12 cells treated with AMB, the mRNA expression of glucose transporters (*GLUT1* and *GLUT4*) and mitochondrial biosynthesis (PGC-1α) was determined. The results showed that *GLUT4*, *GLUT1*, and *PGC-1α* mRNA expression levels were significantly increased after AMB treatment compared with the control nontreated cells (Figure 6).

## 3. Discussion

Hydrogen peroxide (H_2_O_2_) is a reactive oxygen species that has been widely used as an experimental model for inducing oxidative stress-related damage, including in neuronal and skeletal cell models. H_2_O_2_ easily penetrates cell membranes and induces oxidative stress. The toxicity of H_2_O_2_ comes from its activation of NMDA receptors, which increases ROS generation and cell apoptosis [10,13].

Medicinal plants have shown, in many reports, their ability to offer various benefits for human health by their great capability to scavenge ROS. In this study, the protective effect of AMB against H_2_O_2_-induced C2C12 cell death was investigated. Amber is a natural product derived from different types of conifers. More than 40 compounds have been identified in amber, including succinic acid, camphor, borneol, isoborneol, camphene, fenchol, abietic acid, dehydroabietic acid, and isopimaric acid [14]. It has been used in folk medicine for skin allergies, headaches, mental stability, and muscle pain. Mouse skeletal C2C12 cells were the main cell line used to investigate the effects of numerous agents on glucose uptake and *GLUT4* expression in non-differentiated myoblasts and differentiated myotubes [15]. The results of our work revealed that H_2_O_2_ toxicity in C2C12 cells derived from increasing ROS production, ATP depletion, and glucose transporter diminution.

Moreover, different studies have demonstrated that the damage caused by high levels of ROS leads to disequilibrium in cellular signaling pathways, which results in apoptosis and cell death [16]. In addition, with the same dose of H_2_O_2_ (200 uM), Lee et al. [17] demonstrated that hydrogen superoxide induces C2C12 cell death, reaching approximately 40%, which is similar to our results [17]. Our findings showed a significant protective effect of AMB against H_2_O_2_ and oxidative stress. Previous studies confirmed the toxic effect of H_2_O_2_ on C2C12 cells, and they found that after treatment of C2C12 cells with H_2_O_2_, there was an elevation in pro-apoptotic Bax, the Bax/Bcl-2 relative ratio, and the mitochondrial release of cytochrome c [13].

Glucose transporters (GLUTs) regulate cellular glucose uptake. In this study, we focused on *GLUT1* and *GLUT4*, which are the most expressed in muscle cells. *GLUT4* is up-regulated in differentiated muscle cells [18]. Our results showed an overexpression (more than three folds) of *GLUT4* in AMB-treated cells compared with the H_2_O_2_-treated group. The increase in *GLUT4* gene expression explains the rise in glucose uptake levels in differentiated C2C12 cells. The relationship between *GLUT4* mRNA expression and glucose uptake was reported in several works. Alam et al. (2019) demonstrated that the increase in glucose uptake is correlated with the increase in *GLUT4* gene expression in C2C12 cells [6].

Moreover, some studies explained the relationship between ROS and *GLUT1* in different skeletal muscle cells L6 using *GLUT1* inhibitors [19]. Andrisse et al. [19], showed that an increase in ROS levels induced a decrease in *GLUT1* in L6 myoblasts. We demonstrated that amber increases ATP synthesis, reduces ROS production, and enhances glucose uptake in C2C12 cells. These interconnected processes play crucial roles in maintaining muscle cell function and energy homeostasis. In muscle cells, ATP is primarily generated through oxidative phosphorylation in the mitochondria, fueled by the breakdown of glucose and fatty acids. It plays an important role in various regulatory processes including cell signaling, cell proliferation and differentiation, and muscle contraction [20]. ATP is necessary to ensure the normal functioning of the glucose uptake process through the translocation of *GLUT4* and *GLUT1* to the cell surface [20,21]. However, reduced glucose uptake and altered metabolism can lead to increased ROS production and oxidative stress in muscle cells [22]. In addition, mitochondrial dysfunction, often associated with diabetes and oxidative stress, can compromise ATP synthesis and increase ROS production. Hence, impaired ATP synthesis and increased ROS levels can contribute to muscle weakness, fatigue, and other muscle dysfunctions seen in diabetes and oxidative stress conditions.

To investigate the effect of AMB on mitochondrial activities, the mitoTracker green test was conducted and the gene expression of *PGC-1α* was measured. *PGC-1α* enhances oxidative phosphorylation, mitochondrial biogenesis, and the oxygen consumption rate. The data showed an accordance between mitochondrial copy number and *PGC-1α* gene expression. When AMB treatment increased the mitochondrial number, the *PGC-1α* gene expression increased too. The effect of AMB on *PGC-1α* is still not clear yet in our study, however, the mechanism may be an up-regulation of the AMPK pathway which governs the activity of *PGC-1α*. Several studies showed that the activation of *PGC-1α* is linked to the activation of the AMPK pathway [23]. In addition, O’Neill et al. [24] demonstrated that the activation of AMPK promotes glucose uptake and mitochondrial biogenesis in skeletal muscle.

On the other hand, it was reported that *PGC-1α* can regulate mitochondrial network dynamics and mitochondrial ROS in C2C12 cells [25]. In addition, the degradation of *PGC-1α* is associated with mitochondrial dysfunctions. These findings were similar to our results; when amber reduced ROS, *PGC-1α* gene expression was increased and mitochondrial copy number was raised.

## 4. Materials and Methods

### 4.1. Chemical and Reagents

The chemical and reagents used in this study were purchased as follows: MitoTracker Green FM (Invitrogen/Molecular Probes, Eugene, OR 97402, USA); ethanol, glucose assay kit, and 3-(4,5-dimethylthiazol-2-yl)-2,5-diphenyltetrazolium bromide (MTT) (FUJIFILM Wako, Osaka, Japan); DCFDA/H2DCFDA, cellular ROS Assay kit (Abcam, Cambridge, UK); Dulbecco’s modified Eagle medium (DMEM), fetal bovine serum (FBS), and hydrogen peroxide (H_2_O_2_) (Sigma-Aldrich, St. Louis, MO, USA); horse serum (HS) (Gibco, Yokohama, Japan); penicillin and streptomycin (ICN Biomedicals, Tokyo, Japan); and ATP assay reagent (TOYO INK GROUP (Tokyo, Japan).

### 4.2. Sample Preparation

Baltic amber (Kaliningrad, Russia) was crushed, powdered, and extracted twice with 50% ethanol at 40 °C for 1 h. The filtrate was freeze-dried to obtain a powder.

### 4.3. Cell Culture

C2C12 myoblasts were obtained from the RIKEN BioResource Research Center (Tsukuba, Japan). The cells were cultured in Dulbecco’s modified Eagle’s medium (DMEM) supplemented with 10% FBS, penicillin (100 Us/mL), and streptomycin (100 mg/mL), and incubated in an atmosphere of 5% CO_2_ at 37 °C. To induce differentiation, the medium was replaced with DMEM containing 2% horse serum when the cells reached 95% confluency. Experiments were performed in differentiated C2C12 myotubes 5~7 days after differentiation was induced.

### 4.4. Protective Properties of AMB against H_2_O_2_-Induced Cytotoxicity in C2C12 Cells

C2C12 cells were seeded in 96-well plates at a density of 1 × 10^4^ cells/well, allowed to attach for 24 h, and subsequently treated with various concentrations of amber (AMB) (0, 10, 25, 50, 75, and 100 µg/mL) or H_2_O_2_ (50, 100, 150, 200, 250, and 300 µM). After an additional 24 h of incubation, cell viability was evaluated by using 3-(4,5-dimethylthiazol-2-yl)-2,5-diphenyltetrazolium bromide (MTT, 5 mg/mL in PBS). Briefly, 15 µL of MTT solution was added to each well, and the plate was incubated for 6 h at 37 °C in 95% humidified air with 5% CO_2_. Then, 100 µL of 10% (*w*/*v*) sodium dodecyl sulfate (SDS) was added to each well, the plate was incubated overnight, and the absorbance at 570 nm was determined. The control wells, which contained cultured cells with medium only, were considered to have 100% cell viability; otherwise, viability was reported as a percentage of the control value. For each experiment, each treatment was performed in triplicate. To investigate the protective effect of AMB against H_2_O_2_-induced cell death, C2C12 cells were pretreated with AMB at 10, 25, and 50 µg/mL for 24 h, followed by treatment with 200 µM H_2_O_2_ for 24 h, and cell viability was determined as mentioned above.

### 4.5. ROS Determination

The level of intracellular ROS was quantified with the aid of the lipophilic and non-fluorescent compound dihydrodichlorofluorescein diacetate (H2DCF-DA). Inside cells, the acetate moieties of DCFH-DA were cleaved and oxidized, primarily by H_2_O_2_ to green, fluorescent 2′,7′-dichlorofluorescein (DCF). To assess the effect of AMB on the production of ROS in H_2_O_2_-stressed cells, C2C12 cells were seeded at 0.5 × 10^4^ cells/well in 96-well plates and allowed to attach and reach confluence for 24 h. The cells then were differentiated by adding 2% horse serum (HS) for five days and then treated with AMB for 24 h. After 24 h, cells were treated with 100 μL of H2DCF-DA at 25 μM for 60 min and then treated with 150 µM H_2_O_2_ for 60 min or 30 min, after which the fluorescence in each well was measured by using a multi-detection microplate reader (Synergy H1, BioTek, Agano city, Nigata, Japan) at wavelengths of 485/535 nm (excitation/emission). Fluorescence was reported as a percentage of that in the untreated control.

### 4.6. Glucose Uptake

The glucose level in the cell culture medium was determined by a calorimetric method using a LabAssayTM glucose kit (Wako Pure Chemical Industries, Ltd., Osaka, Japan).

C2C12 myoblasts were seeded and grown on 24-well plates. After 5 days of differentiation in a 2% HS culture medium, the formation of myotubes was well defined. The myotubes were treated with AMB samples for 24 h, and the glucose in the medium was measured every 6 h as described in the manufacturing protocol. In brief, 2.5 μL of medium from each well was taken and then, 200 μL of glucose reagent was added. After 15 min of incubation at 37 °C, the optical density (O.D.) was measured at 505 λ.

### 4.7. Intracellular ATP Measurement

Intracellular ATP levels were measured based on the luciferin–luciferase system. C2C12 cells were seeded at 0.5 × 10^4^ cells/well in 96-well plates and allowed to attach and reach confluence for 48 h at 37 °C. After 5 days of differentiation, cells were treated with AMB at different doses: 10, 25, and 50 μg/mL for 6 and 12 h. Then, ATP was measured according to the manufacturing kit with small modifications. In brief, 100 μL of ATP extract was added to cells for 5–10 min at room temperature. Subsequently, 100 μL of ATP lighting was added to the cell lysate. Finally, the luminescence in each well was measured by using a multi-detection microplate reader (Synergy H1, BioTek, Japan) at 480/530 nm (excitation/emission), and the activity was reported as a percentage of that of the untreated control.

### 4.8. MitoTracker Green

The mitochondrion is a key regulator of the metabolic activity of a cell, including the production and degradation of free radicals. MitoTracker green (MTG) is a fluorescent probe that has been commonly used to assess mitochondrial mass (or mitochondrial copy number). It is expected that this dye selectively accumulates in the mitochondrial matrix where it covalently binds to mitochondrial proteins by reacting with the free thiol groups of cysteine residues [26].

C2C12 cells were seeded at 0.5 × 10^4^ cells/well in 96-well plates and allowed to attach and reach confluence for 48 h at 37 °C. After 5 days of differentiation with 2% HS, cells were treated with AMB at different doses: 10, 25, and 50 μg/mL for 48 h. After treatment, the cells were incubated in prewarmed 100 nM MitoTracker Green FM (Invitrogen/Molecular Probes, Eugene, OR 97402, USA) at 37 °C for 30 min. The cells were then washed, and fresh and warmed buffer was added for live cell imaging and fluorescence measurement using a plate reader (Ex 485/Em 535). Images were captured using a fluorescence microscope (KEYENCE, BZ-X810, Osaka, Japan).

### 4.9. Gene Expression Analysis

C2C12 cells were plated at 20 × 10^4^ cells/well in a 6 cm dish and allowed to attach and proliferate for 48 h, and then the medium, containing 2% HS, was changed every day over 7 days of differentiation. After becoming fully differentiated, cells were treated with amber at 25 and 50 µg/mL for 24 h. Total RNA was extracted from C2C12 cells using RNAiso Plus (TaKaRa Bio Inc., Kusatsu, Shiga, Japan). RNA was quantified by Thermo Scientific Nano 2000 (San Diego, CA, USA). cDNA was synthesized using a PrimeScript™ RT Reagent Kit with a gDNA Eraser (TaKaRa Bio Inc., Kusatsu, Shiga, Japan).

Quantitative PCR was performed on the cDNA obtained from amber-treated C2C12 cells. Each cDNA was amplified using specific primers (at 50 °C for 2 min, 95 °C for 10 min, 95 °C for 15 s, 60 °C for 15 s, for 40 cycles). The internal standard was *GAPDH* [27]. Primer sets used in the analysis were as follows: glycelaldehyde 3-phosphate dehydrogenase (*gapdh*) set: forward 5′TGGTGAAGGTCGGTGTGAACGG3′, reverse 5′TGCCGTTGAATTTGCCGTGAGT3′; glucose transporter 1 (*glut1*) set: forward 5′CCAGCTGGGAATCGTCGTT3′, reverse 5′CAAGTCTGCATTGCCCATGAT3′; glucose transporter 4 (*glut4*) set: forward 5′TGCTGGGCACAGCTACCC3′, reverse 5′CGGTCAGGCGCTTTAGAC3′; peroxisome proliferator-activated receptor gamma coactivator 1-alpha (pgc-1α) set: forward 5′ACCATGACTACTGTCAGTCACTC3′, reverse 5′GTCACAGGAGGCATCTTTGAAG3′. Gene expression was normalized to *GAPDH* and reported as a fold change compared to the control.

### 4.10. Statistical Analysis

The results are expressed as mean ± SD. Comparisons between groups were made using Student’s *t*-tests. Differences were considered significant at a *p*-value < 0.05.

## 5. Conclusions

Our data show a significant protective effect of amber extract against H_2_O_2_-induced C2C12 cell death. In addition, amber extract improves glucose uptake and ATP production in the myotubes through the up-regulation of the mitochondrial biogenesis-regulating factor, *PGC-1α*, and the glucose transporters *GLUT4* and *GLUT1*, and the down-regulation of ROS generation. Thus, this finding suggests that amber extract, by improving energy metabolism and mitochondrial biogenesis, has beneficial effects on the promotion of skeletal muscle function. However, the precise mechanism by which amber enhanced glucose uptake, improved ATP production, and decreased ROS levels in C2C12 cells was not fully understood. Further experiments are needed to understand how amber protects C2C12 cells against H_2_O_2_. First, it is necessary to elucidate the protective effect of amber on pro-apoptotic Bax and anti-apoptotic Bcl-2. Second, an in vivo evaluation of amber’s effect on muscle atrophy and physical fatigue is needed to confirm the beneficial effect of amber on ATP production, ROS clearance, and glucose uptake.

## Data Availability

Data is contained within the article.
